# The Role of Interleukin-6 Family Members in Cardiovascular Diseases

**DOI:** 10.3389/fcvm.2022.818890

**Published:** 2022-03-23

**Authors:** Yongqi Feng, Di Ye, Zhen Wang, Heng Pan, Xiyi Lu, Menglong Wang, Yao Xu, Junping Yu, Jishou Zhang, Mengmeng Zhao, Shuwan Xu, Wei Pan, Zheng Yin, Jing Ye, Jun Wan

**Affiliations:** ^1^Department of Cardiology, Renmin Hospital of Wuhan University, Wuhan, China; ^2^Cardiovascular Research Institute, Wuhan University, Wuhan, China; ^3^Hubei Key Laboratory of Cardiology, Wuhan, China

**Keywords:** cardiovascular diseases, IL-6 family cytokines, atherosclerosis, coronary artery disease, cardiac remodeling

## Abstract

Cardiovascular disease is one of the main causes of human mortality. Cytokines play crucial roles in the development of cardiovascular disease. Interleukin (IL)-6 family members are a series of cytokines, including IL-6, IL-11, IL-30, IL-31, OSM, LIF, CNTF, CT-1, CT-2, and CLC, that regulate multiple biological effects. Experimental and clinical evidence shows that IL-6 family members are closely related to cardiovascular diseases such as atherosclerosis, hypertension, aortic dissection, cardiac fibrosis, and cardiomyopathy. This review mainly discusses the role of IL-6 family members in cardiovascular disease for the sake of identifying possible intervention targets for cardiovascular disease prevention and treatment.

## Introduction

Currently, cardiovascular diseases (CVDs) are the leading cause of human death and morbidity worldwide. They not only threaten the safety and quality of life of patients but also place a heavy burden on society (1, 2). Inflammation plays an important role in CVD, and markers of inflammation can predict future CVD events (3).

The interleukin-6 family comprises IL-6, IL-11, IL-30, IL-31, and non-IL molecules, including oncostatin M (OSM), leukemia inhibitory factor (LIF), ciliary neurotrophic factor (CNTF), cardiotrophin 1 (CT-1), and cardiotrophin-like cytokine (CLC). They are characterized by sharing the common receptor subunit glycoprotein 130 (gp130) and sharing the structure of four-helices with an up-up–down-down topology.

A large number of studies have confirmed that IL-6 has both proinflammatory and anti-inflammatory effects via different IL-6Rs. The receptor complexes of IL-6 are composed of IL-6R or soluble IL-6R and gp130. It seems that the proinflammatory effect mainly relies on trans-signaling mediated by sIL-6R and that the anti-inflammatory effect mainly depends on membrane-bound IL-6R (4–7). IL-6 induces Th17 differentiation, suppresses Treg differentiation, and stimulates the polarization of M2 macrophages (8–10). Lymphocytes, monocytes/macrophages, adipocytes, and hematopoietic and endothelial cells are the cellular sources of IL-6 (11). The gp130 protein is expressed in almost all tissues (12).

IL-11 is reported as a pro- and anti-inflammatory cytokine. The signal transduction process of IL-11 is similar to that of IL-6, and the IL-11/IL-11R complex needs to be formed before gp130 can be activated. Additionally, there are both classic and trans-signaling pathways through IL-11R or sIL-11R complexes (13, 14). The cellular sources of IL-11 are T cells, B cells, macrophages, cardiac myocytes, etc. The main source of IL-11 is not clear. It can induce Th2 and Th17 differentiation, suppress Th1 differentiation and inhibit macrophage activity (13).

IL-30 is the p28 subunit of IL-27 but has some functions that are independent of IL-27. IL-30 is a natural antagonist of gp130, so IL-30 may offer a therapeutic strategy against inflammation (15, 16). IL-30 has been shown to inhibit the differentiation of Th1 and Th17 cells (17). IL-30 is secreted by activated macrophages and splenocytes (18).

IL-31 is a proinflammatory cytokine that activates the receptor complex of IL-31RA and OSMR. IL-31 induces Th1 and inhibits Th17 differentiation *in vitro* (19, 20). IL-31 is secreted by T cells and granulocytes, especially Th2 cells (21).

OSM has been shown to bind to both the gp130/OSMR complex and gp130/LIFR complex and shows proinflammatory effects (22–24). *In vitro* experiments have shown that OSM inhibits the proliferation of Th17 cells and induces dendritic cell (DC) maturation and Th1 polarization (25, 26). It is secreted by activated monocytes/Møs, DCs, neutrophils, T lymphocytes, and hematopoietic cells in the bone marrow (22).

LIF is an anti-inflammatory cytokine that binds to the gp130/LIFR complex (27). LIF is highly produced by Treg cells in both humans and mice. LIF inhibits inflammation by promoting Treg differentiation and inhibiting Th17 cell differentiation (28).

CNTF binds to CNTFR and then induces heterodimerization of gp130 and LIFR, which is involved in signal transduction (29). The cellular source and its role in the immune response remain to be studied.

CT-1 plays an anti-inflammatory role and binds to the complex of gp130 and LIFR and possibly requires the CT-1R subunit in neuronal cells (30, 31). CT-1 mRNA is expressed in the adult human heart, skeletal muscle, ovary, colon, prostate, and testis. CT-1 is mainly secreted by cardiac nonmyocytes in the heart. However, the cellular source still needs to be studied (31–33).

CLC or the heterodimeric cytokine cardiotrophin-like cytokine:cytokine-like factor-1 (CLC:CLF-1) binds to CNTFR and then interacts with gp130/LIFR, which subsequently has a proinflammatory role (34–36). Evidence has shown that CLC is secreted by circulating lymphocytes and can stimulate B cells, activate Møs, and promote monocyte numbers (37–39).

The signaling pathways of IL-6 family members are similar but distinct because of their similar but distinct receptor complexes. One major signaling pathway is the activation of Janus kinase (JAK) tyrosine kinase family members, leading to the activation of the signal transducers and activators of transcription (STAT) transcription factors, mostly STAT3. Another major signaling pathway is the JAK-SH2 domain tyrosine phosphatase 2 (SHP2)-mitogen-activated protein kinase (MAPK) pathway (23, 40–43). The detailed pathways are illustrated in Table 1.

**Table 1:** The receptor complexes and pathways of IL-6 family members.

	**Receptors complexes**	**Pathways**	**Role in immune response**	**Cellular source**	**References**
IL-6	IL-6R/sIL-6R+gp130	JAK1,JAK2,TYK2, STAT3,STAT1, MAPK, PI3K	Induce Th17 differentiation Suppress Treg differentiation stimulate the polarization of Mø	Lymphocytes, monocytes/ Mø, adipocytes, hematopoietic and endothelial cells	(5–11, 44–47)
IL-11	IL-11R/ sIL-11R +gp130	JAK, STAT3, MAPK, PI3K	Induce Th2, Th17 differentiation Suppress Th1 differentiation Inhibit Mø activity	T cells, B cells and other cell types Main source is unclear	(13, 14, 48)
IL-30	gp130	STAT1,STAT3, MAPK	Inhibit Th1,Th17 differentiation	activated Mø splenocytes	(15–18)
IL-31	IL-31RA+OSMR	STAT1, STAT3, STAT5, PI3K, MAPK	Indude Th1, inhibit Th17 differentiation	Th cells	(19, 21)
OSM	gp130+OSMR or gp130+LIFR	JAK,STAT, MAPK, PI3K, PKCδ	Inhibit Th17 activation induces dendritic cell maturation and Th1 polarization	Activated Monocytes/Mø, DCs, neutrophils,T-lymphocytes. Hematopoietic cells in the bone marrow	(22, 23, 25, 26)
LIF	gp130+LIFR	JAK1,JAK2, TYK2, STAT1,STAT3,STAT5, PI3K, MAPK	Prompt Treg differentiation Inhibit Th17 differentiation	Tregs	(27, 28)
CNTF	CNTFR+gp130+LIFR	JAK, STAT1,STAT3, MAPK, PI3K	-	-	(29, 49, 50)
CT-1	LIFR+gp130 or LIFR+gp130+CT-1R	JAK1, JAK2, TYK2, STAT1, STAT3, STAT5, MAPK, PI3K	Inhibit M1 polarization Prompt M2 polarization	Cardiac nonmyocytes	(30–33)
CLC	CNTFR+LIFR+gp130	JAK1, JAK2, TYK2, STAT1, STAT3, STAT5, MAPK, PI3K	Stimulate B cell Activate Mø Promote monocytes number	circulating lymphocytes	(34, 35, 37–39)

*Mø, macrophages; DC, dendritic cell*.

## Interleukin-6 Family Members and Cardiovascular Disease

Increasing evidence demonstrates that inflammation plays an important role in the development of cardiovascular disease (51–54). IL-6 family members modulate the immune response and inflammatory activity and then participate in the development of cardiovascular diseases (41, 55, 56).

### Interleukin-6 Family Members and Atherosclerosis, Coronary Artery Disease

Atherosclerosis is the leading cause of coronary artery disease (CAD). It causes life-threatening events such as thrombosis as well as the rupture or erosion of atherosclerotic plaques (57). Atherosclerosis is a chronic inflammatory disease, so many researchers have focused on the potential mediators that initiate and maintain this vascular disease (58).

The progression of carotid atherosclerosis is positively correlated with the elevation of IL-6 (59). IL-6 plays an important role in regulating the downstream inflammatory responses that contribute to the development of atherosclerosis (60, 61). IL-6 perpetuates vascular inflammation by promoting smooth muscle cell (SMC) proliferation and migration, endothelial dysfunction and the recruitment and activation of inflammatory mediators, which result in atherosclerotic plaque development and plaque destabilization (61, 62). Higher IL-6 measured at 24 h after ST-elevation myocardial infarction (STEMI) is associated with a larger infarct size and diminished cardiac function measured at 4 months. IL-6 can be a potential biomarker for STEMI prognosis and a target for improving prognosis (63). Clinical data show that IL-6 is a biomarker of mortality from unstable CAD (64). The increase in IL-6 levels has a strong relationship with future cardiac events and CAD mortality in anginal syndrome or healed myocardial infarction patients (65). The use of tocilizumab, an IL-6 receptor antagonist, reduces the inflammatory response in non-STEMI (NSTEMI) patients, which may be beneficial to patients but still needs further study (66). Canakinumab is a monoclonal antibody against IL-1β and can modulate the IL-6 pathway to decrease the major adverse cardiovascular event (MACE) rate (67). On the one hand, experimental atherosclerosis studies show that treatment with recombinant IL-6 (rIL-6) promotes early atherosclerosis in C57Bl/6 and ApoE-deficient mice. The rIL-6-treated mice showed higher plasma levels of proinflammatory cytokines such as TNFα and IL-1β, which can promote the development of fatty streaks by enhancing the accumulation of foam cells. In addition, proinflammatory cytokines can activate macrophage-monocytes so that cell migration into the intima, lipid uptake, and low-density lipoprotein (LDL) oxidation are increased (68). On the other hand, IL-6 has an atheroprotective effect because lifetime IL-6 deficiency leads to more severe atherosclerosis rather than inhibition of plaque formation. It is believed that lifetime deficiency of IL-6 breaks the balance of IL-6 and IL-10 and thus promotes the development of atherosclerosis (69, 70). OSM is expressed in atherosclerotic lesions and promotes SMC proliferation, migration and extracellular matrix synthesis, which may contribute to atherosclerosis progression. OSMR-β deficiency alleviates atherosclerosis and plaque instability. Serum OSM levels are elevated in CAD patients compared to those without CAD (71–73). Nevertheless, chronic administration of OSM can attenuate the development of plaques and improve plaque severity in APOE^*^3Leiden.CETP mice. The possible mechanisms might involve regeneration of the endothelial barrier, induction of SMC proliferation, and a reduction in the inflammatory Ly-6CHigh monocyte subset. Patients with higher serum OSM have increased post incident coronary heart disease survival probability (74). Because OSM can activate both gp130/OSMR receptor complex and gp130/LIFR receptor complex. The selective inhibition of OSMR-β might be a potential therapeutic target. Further study is needed. In a rabbit model, LIF can retard the progression of atherosclerosis because it can reduce macrophages in the neointima of uninjured arteries and can regulate iNOS activity to maintain beneficial levels of nitric oxide (NO) (75, 76). CT-1 promotes the development of atherosclerotic lesions because it can induce the migration and proliferation of vascular smooth muscle cells and collagen-1 production. It can stimulate inflammatory responses, and the formation of foam cells is correlated with CD36 and ACAT1 upregulation in macrophages (77). ApoE and CT-1 double knockout (DKO) mice have smaller atherosclerotic lesions than ApoE KO mice. CT-1 deficiency induces atheroprotective immune cells, including Bregs, Tregs and B1a cells. Moreover, CT-1 deficiency is beneficial to plaque stability because DKO mice have an increased collagen content in the aortic sinus, a significant reduction in MMP9 expression and necrotic core area and an increase in the fibrous cap thickness in atherosclerotic roots. The present study demonstrates the inhibition of CT-1 attenuates atherosclerosis progression and development. But the application in patients still needed to be studied (78). Cardiotrophin-like cytokine factor 1 (CLCF1) upregulates scavenger receptor A 1 (SR-A1) expression, which is the major mechanism of the increase in lipoprotein uptake, inducing the formation of macrophage-foam cells. A murine experiment indicated that SR-A1 deficiency decreased atherosclerotic lesions (44, 79). Kim, Jun W et al. have engineered CLCF1 variants that can inhibit or activate CNTFR. The application of it in atherosclerosis might be meaningful research (80).

### Interleukin-6 Family Members and Hypertension

Hypertension is a leading cause of cardiovascular events, which contributes greatly to mortality and disability. With the increased understanding of immunology, evidence that the immune system may lead to hypertension is increasing (81).

The inhibition of IL-6 attenuates the development of salt-sensitive hypertension in rat models. IL-6 KO mice have a lower mean arterial pressure (MAP) than WT mice. The deletion of IL-6 can prevent the activity of the JAK2/STAT3 pathway, which plays a role in Ang II-induced hypertension (82–84). The circulating levels of IL-6 have a positive relationship with blood pressure (85). Clinical data indicates that the hypomethylation of the IL-6 gene promoter may increase the risk of essential hypertension by upregulating the expression of IL-6 (86). CT-1 is significantly increased in untreated hypertensive patients compared with normotensive subjects (87, 88). SA study showed that excess CT-1 may contribute to inappropriate left ventricular growth in hypertension patients (89). Research on other IL-6 family members associated with hypertension remains to be conducted. Aortic stiffness is measured by pulse wave velocity (PWV) and can predict cardiovascular morbidity and mortality in hypertension patients. Clinical data shows a positive relationship between IL-6 and PWV (90–93). Du, Bing et al. showed that the LDLr-/- mice had larger PWV and *ex vivo* intrinsic mechanical properties, which means that LDLr-/- mice had arterial stiffness. IL-6 from aortic perivascular adipose tissue (PVAT) plays a critical role in promoting arterial stiffness. What's more, the inhibition of IL-6 can attenuate arterial stiffness, and the treatment of IL-6 can aggravate (94).

### Interleukin-6 Family Members and Aortic Aneurysms and Aortic Dissection

Aortic aneurysms are dilations of the aorta larger than 50% of the normal aorta diameter (95). Abdominal aortic aneurysms (AAAs) and thoracic aortic aneurysms (TAAs) are the most common aortic aneurysms (96). Acute aortic dissection (AD) is a rare disease but has high mortality. The blood penetrates the aortic wall layers and creates a so-called false lumen (FL), which is a cavity within the medial layer. The FL and true lumen (TL) are separated by dissection membranes. The rupture of the FL or a second tear in the dissection membrane would cause serious consequences (97).

The expression of IL-6 is increased in β-aminopropionitrile (BAPN)-induced AD rat models. Circulating plasma IL-6 levels are elevated in AAA patients. Experimental data support that aortic aneurysms can secrete IL-6 (98–100). Paige et al. found that selective inhibition of the IL-6 trans-signaling pathway can decrease aortic rupture and death in 2 AAA mouse models, which shows us a potential therapeutic target for AAA (101). The expression of IL-6 is increased in AD rat models. IL-6 may enhance the expression of MMP-2 and may promote extracellular matrix degradation of the vascular wall, which promotes the formation of AD (98). Lv, Xiao-Chai et al. found that plasma IL-6 level is elevated in postoperative delirium (POD) patients after aortic dissection surgery. Thus, plasma IL-6 values can be used to evaluate AAD patients' POD outcomes (102). Besides, the high level of IL-6 and D-dimer has predictive value in assessing the poor prognosis after acute Stanford type A aortic dissection surgery (103). IL-11 is significantly increased in thoracic AD and can be a potential biomarker for AD (104). OSM is a proinflammatory mediator and is upregulated in abdominal AD patients. Thus, it may contribute to the development of aortic aneurysms (105, 106). The level of CT-1 is higher in AAA tissues. CT-1 can stimulate aortic endothelial cells to overproduce MMP-1, which leads to ECM degradation. These mechanisms are associated with the formation and progression of AAA (107, 108).

### Interleukin-6 Family Members and Cardiac Remodeling

Cardiac fibrosis is characterized by the excessive deposition of extracellular matrix (ECM) proteins that results in the expansion of the cardiac interstitium, which is a common pathophysiologic companion of most myocardial diseases. It is related to cardiac dysfunction, arrhythmogenesis, and adverse outcomes (109, 110). Cardiomyopathy is a disease that weakens the heart muscle, attenuating the heart's ability to pump blood and possibly leading to heart failure (HF) (111). HF is a complex clinical syndrome that is caused by structural or functional impairment of ventricular filling or ejection of blood (112). Proinflammatory cytokines trigger a series of pathological responses, such as oxidative stress, endothelial dysfunction, induction of myocyte apoptosis, and hypertrophy, which ultimately leads to cardiomyocyte dysfunction (113).

An experimental study showed that IL-6 plays a central role in myocardial fibrosis that depends on the activation of the MAPK and CAMKII-STAT3 pathways. IL-6 is a downstream signal of hypoxia-induced mitogenic factor (HIMF), and its inhibition can prevent fibroblast activation (114). In addition, the overexpression of IL-6 increases TGF-β1-mediated MMP2/MMP3 signaling to induce myofibroblastic proliferation, differentiation, and fibrosis (115). IL-6 KO mice had a lower degree of cardiac fibrosis. Thus, anti-IL-6 can be a potential therapeutic target for decreasing cardiac fibrosis (116). The expression of IL-11 is positively related to myofibroblast numbers and is higher in mice with cardiac fibrosis than in wild-type mice (117). Anti-IL-11 treatment can attenuate the profibrotic effect on the heart of transverse aortic constriction (TAC) mouse model (118). Interestingly, Obana et al. found that IL-11 attenuates cardiac fibrosis in mouse models after myocardial infarction through the activation of STAT3 (119). Thus, when faced with different diseases, the appropriate application of IL-11 or its antagonist is a potential therapeutic target and needs further investigation. The antifibrotic effects of OSM are achieved by inhibiting the TGF-β1-mediated activation of cardiac fibroblasts in TAC mouse models (120). LIF cDNA injection was found to attenuate cardiac fibrosis in mice after myocardial infarction (121). Chronic administration of LIF improves the heart function of mice (122). Therefore, LIF may be a novel treatment for cardiac fibrosis. López et al. found that CT-1 can be a biomarker of myocardial fibrosis (123). CT-1 is believed to promote the development of cardiac fibrosis by upregulating Gal-3 through the ERK 1/2 and STAT3 pathways (124).

Clinical data have demonstrated that idiopathic dilated cardiomyopathy patients with higher serum IL-6 have a lower ejection fraction and worse prognosis (125). Serum IL-6 concentration is increased in patients with takotsubo cardiomyopathy (126). IL-6 KO mice with dilated cardiomyopathy showed better cardiac function and less myocardial cell apoptosis than WT mice with dilated cardiomyopathy because of the inhibition of STAT3 (127). The inhibition of IL-6/STAT3 signaling pathway may offer a new target for cardiomyopathy. Diabetic cardiomyopathy mice exhibited increased OSM. Moreover, OSM-treated diabetic mice exhibit worse cardiac function. Knockout of the OSM receptor Oβ attenuated dilated cardiomyopathy injury by inhibiting the B-Raf/MEK/ERK cascade (128). OSM is consistently upregulated in dilated cardiomyopathy patients and mouse models. OSM protects the damaged myocardium by inducing dedifferentiation. However, prolonged stimulation with OSM prompts the progression of HF in dilated cardiomyopathy (129). The plasma levels of CT-1 are increased in hypertrophic cardiomyopathy and are associated with the severity of left ventricular hypertrophy (130). The expression of CT-1 is increased in the acute stage of Chagas disease (131). The plasma level of CT-1 is increased in dilated cardiomyopathy patients with congestive HF compared to control subjects (132).

An observational study showed that higher IL-6 plasma levels were found in half of HF patients and were associated with reduced left ventricular ejection fraction (LVEF), atrial fibrillation, and poorer clinical outcomes (133, 134). Genetic deletion of IL-6 alleviates left ventricular dysfunction through the STAT3 pathway in a transverse aortic constriction-induced pressure overload-HF mouse model (135). Moreover, the inhibition of IL-6/STAT3 by raloxifene can attenuate inflammation in the same model (136). Higher plasma IL-11 levels predict poor outcomes in HF patients (137). Plasma OSM levels are elevated in HF patients with reduced ejection fraction (HFrEF) (138). Kubin, Thomas et al. found that OSM is the key modulator of HF that induces cardiomyocyte dedifferentiation and contractility loss through the MAPK cascade in a mouse model of left anterior descending coronary artery (LAD) ligation (129). LIF mRNA is elevated in the left ventricle of congestive HF patients, and the circulating LIF level is increased with the deterioration of congestive HF (139, 140). Moreover, the upregulation of LIF in the ventricle was reproduced in the Dahl salt-sensitive (DS) rat chronic HF model (141). Myocardial and circulating CT-1 levels are increased in HF patients and are positively correlated with HF patient mortality, which can be used as a biomarker for determining prognosis (124, 142–144). CT-1 upregulates galectin-3 (Gal-3) via the ERK 1/2 and STAT3 pathways to promote cardiac fibrosis and hypertrophy, which are involved in the development of HF (124). López, Natalia et al. illustrate that LIFR is downregulated in spontaneously hypertensive rats HF model that attenuates the cytoprotection of CT-1. The upregulation of LIFR might be a potential target (145).

### Interleukin-6 Family Members and Atrial Fibrillation

Atrial fibrillation (AF) is the most common cardiac arrhythmia and leads to detrimental consequences. Increasing evidence supports that inflammation plays a crucial role in the pathophysiology of AF (146, 147). Thus, the process of inflammation is a potential therapeutic target for AF.

Amdur et al. found that elevated levels of IL-6 are associated with an increased risk of AF in chronic kidney disease (CKD) patients, which suggests that IL-6 can serve as an inflammatory biomarker for AF in CKD patients (148). Also, IL-6 levels are associated with AF in CAD patients (149). Elderly patients who received recombinant human IL-11 were observed to have an increased incidence of AF (150). OSM is increased in atrial tissue of AF patients with thrombus (151). Patients with higher levels of CT-1 have more frequent AF relapse (152). The correlation between the IL-6 family and AF still needs further study.

### Interleukin-6 Family Members and Myocarditis

Myocarditis is an uncommon but potentially life-threatening heart disease (153). Myocarditis induces a broad range of pathological immune processes in the heart, which causes structural and functional abnormalities (154).

IL-6 plays a key role in the development of autoimmune heart disease, possibly by upregulating complement C3. IL-6 KO mice with autoimmune myocarditis showed a reduction in inflammatory responses, the proliferation of autoreactive CD4+ T cells, and the expression of ICAM-1 and VCAM-1, which reduced myocarditis susceptibility (155). IL-6 is crucial for Th17 differentiation through the induction of retinoic acid receptor-related orphan nuclear receptor, which is a critical event in the onset of experimental autoimmune myocarditis (EAM). The blockade of IL-6R inhibits the initiation of EAM (156). Adequate levels of IL-6 attenuate the damage from viral infection in the early stage of inflammation. Nevertheless, overexpression of IL-6 aggravates viral myocarditis (157). CT-1 is expressed in cardiac myocytes infected with Coxsackievirus B3 (CVB3) and induces pathologic responses in acute myocarditis. However, the early expression of CT-1 might have a protective effect on cardiac myocytes by inhibiting TNF-α and IL-1α expression (158).

### Interleukin-6 Family Members and Cardiac Ischemia Reperfusion Injury

Reperfusion of the myocardium can induce further cardiomyocyte apoptosis after cardiac ischemia, such as occurs with myocardial infarction or heart transplantation (159). Many studies have shown that myocardial apoptosis mediated by inflammation is one of the crucial processes of ischemia-reperfusion (I/R) injury (160, 161).

IL-6 prompts the development of infarction after cardiac I/R injury, whereas IL-6 deficiency attenuates I/R injury. However, the beneficial effects cannot be explained by modification of other inflammatory mediators, coagulation activation, or neutrophil influx. The related mechanisms need to be further explored (162). The administration of IL-11 has a protective effect on the heart from I/R injury via the STAT3 pathway. Thus, it can be a potential therapeutic target against I/R injury (163, 164). OSM is thought to be an important factor for tissue repair after cardiac I/R injury because it upregulates monocyte-chemoattractant-protein (MCP-1) expression and stimulates the proliferation of fibroblasts (165). Experimental data show that OSM protects the heart against cardiac I/R injury through the regulation of mitochondrial biogenesis, cardiomyocyte apoptosis, and insulin sensitivity in diabetic mice (166). OSM can alleviate cardiac dysfunction and reduce the infarct size in mice partly through the Notch3/Akt and AMPK/PGC-1α pathways (167). Pretreatment with LIF has a protective effect on the heart against cardiac I/R injury (168). The significant protective effect that CT-1 has on the heart after I/R injury depends on the activation of the p42/p44 MAPK pathway (169, 170).

### Interleukin-6 Family Members and Other Cardiovascular Diseases

IL-6 family members are also associated with other cardiovascular diseases, such as ventricular fibrillation, congenital heart disease (CHD), and vascular calcification.

Elevated IL-6 serum levels are correlated with the occurrence of spontaneous ventricular tachyarrhythmia and ventricular fibrillation in implantable cardioverter-defibrillator (ICD)-recipient patients with CAD and idiopathic dilated cardiomyopathy (171). The IL-6 variant rs1800795 is associated with CHD among Chinese Han people (172). Moreover, serum IL-6 levels are higher in CHD groups than in control groups (173, 174). Myocardial IL-11 and IL-6 levels are elevated in CHD children and downregulate the microRNA-199a-5p-mediated unfolded protein response through the STAT3 pathway (175). CT-1 is induced in CHD patients and is negatively correlated with arterial oxygen saturation (176). Clinical data show serum IL-6 levels are elevated in hemodialysis patients and chronic kidney disease patients with vascular calcification (177–180). IL-6 promotes vascular calcification by inducing the differentiation of VSMCs into osteoblast-like cells. IL-6/STAT3 pathway upregulates *RUNX2* gene expression, which is an important transcription factor of the differentiation of osteoblast (181). The activation of IL-6-mediated receptor activator of NF-κB ligand(RANKL) plays a crucial role in the development of vascular calcification. And the anti-IL-6 treatment can reduce the SMC calcification (182–184). Moreover, Lee, Guan-Lin et al. also showed the inhibition of IL-6 can attenuate the VSMC calcification (185).

## Conclusion

**Figure 1:**
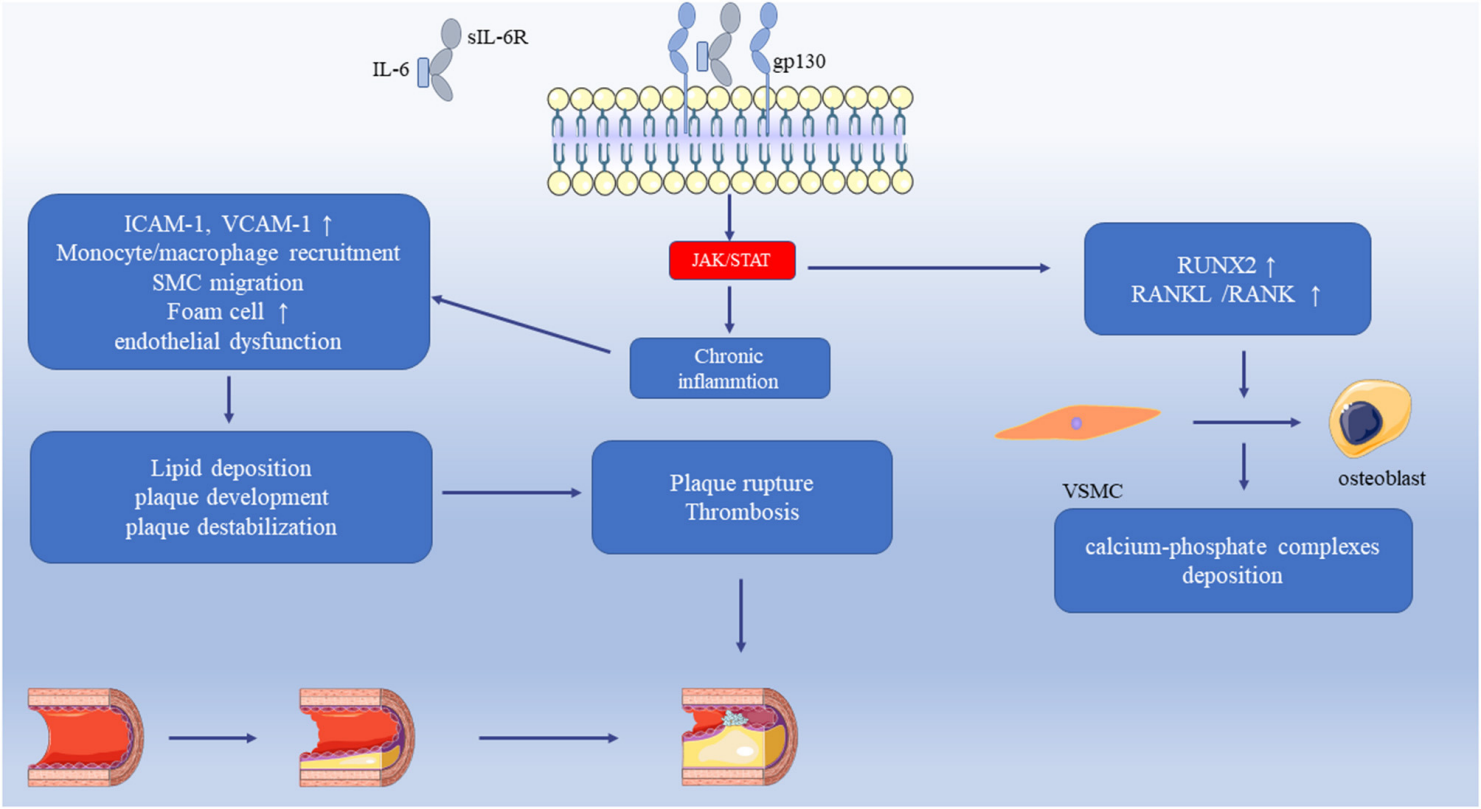
The relationship between IL-6 and atherosclerosis, MI, and vascular calcification. The IL-6 trans-signaling activates the JAK/STAT pathway that leads to chronic inflammation. It increases the adhesion molecules in the vasculature, endothelial dysfunction, the recruitment of monocytes/macrophages, and SMC migration. Monocytes uptake LDL and transform into foam cells that accelerate the progression of atherosclerosis. These pathological processes cause lipid deposition, plaque development, and plaque destabilization. With increasing severity of atherosclerosis, the plaque ruptures, and thrombosis result in myocardial infarction. IL-6 upregulates RUNX2 and RANKL/RANK, which induces the differentiation of VSMC to osteoblast and then induces the calcium-phosphate complexes deposition. JAK, Janus Kinase; STAT, Signal transducers and activators of transcription; LDL, Low-density lipoprotein; RUNX2, Runt-related transcription factor 2; RANKL, Receptor activator of NF-κB ligand; SMC, Smooth muscle cell; VSMC, Vascular smooth muscle cell.

This review describes the molecular receptors of the IL-6 family members, related signaling pathways, and their role in the regulation of inflammation. IL-6 family signal transduction is similar and is dominantly mediated by STAT3. The expression and regulation of IL-6 family members in cardiovascular disease are summarized in Tables 2, 3. Some family members, especially IL-6, have both pro- and anti-inflammatory effects through different receptors and pathways. Thus, the selective inhibition of trans-signaling rather than global inhibition might be a future therapeutic strategy. Cytokines affect the progression of cardiac pathology by regulating complex signaling networks. We illustrated the relationship between IL-6 and atherosclerosis, MI, and vascular calcification in Figure 1. Further research is needed to discover potential therapeutic targets and biomarkers for cardiovascular disease.

**Table 2:** The expression of IL-6 family members on cardiovascular diseases.

**Disease**	**IL-6**	**IL-11**	**OSM**	**CT-1**	**Reference**
AS	Increase	-	Increase	Increase	(59, 71, 78)
CAD	Increase	-	Increase	-	(63, 73)
Hypertension	Increase	-	-	Increase	(85, 87, 88)
AA, AD	Increase	Increase	Increase	Increase	(98–100, 104, 105, 108).
Cardiomyopathy	Increase	-	increase	Increase	(126, 129, 131, 132)
AF	Increase	Increase	Increase	Increase	(148, 151, 152)
Myocarditis	-	-	-	Increase	(158)

*AS, atherosclerosis; CAD, coronary artery disease; I/R, ischemic-reperfusion; AA, arterial aneurysm; AD, aortic dissection; AF, atrial fibrillation*.

**Table 3:** Regulation of IL-6 family members on cardiovascular disease.

**Disease**	**IL-6**	**IL-11**	**OSM**	**LIF**	**CT-1**	**CLC**	**Reference**
AS	Aggravate	-	Controversial	Alleviate	Aggravate	Aggravate	(44, 60, 71, 74, 75, 77)
CAD	Aggravate	-	Alleviate	-	-	-	(65, 74)
Hypertension	Aggravate	-	-	-	Aggravate	-	(83, 89, 135)
AA, AD	Aggravate	-	Aggravate	-	Aggravate	-	(98, 101, 105–108)
Cardiac fibrosis	Aggravate	Controversial	-	Alleviate	Aggravate	-	(114–117, 121, 122, 124)
Myocarditis	Aggravate	-	-	-	Controversial		(155, 156, 158)
Cardiomyopathy	-	-	-	-	Controversial		(129)
I/R	Aggravate	Alleviate	Alleviate	Alleviate	Alleviate		(162–170)

*AS, atherosclerosis; CAD, coronary artery disease; I/R, ischemic-reperfusion; AA, arterial aneurysm; AD, aortic dissection; AF, atrial fibrillation*.

## Author Contributions

YF and DY wrote this article. ZW, HP, XL, MW, YX, JYu, JZ, MZ, SX, WP, and ZY searched the literature. JYe and JW provided ideas and financial support. All authors contributed to the article and approved the submitted version.

## Funding

This work was supported by grants from National Natural Science Foundation of China (82070436).

## Conflict of Interest

The authors declare that the research was conducted in the absence of any commercial or financial relationships that could be construed as a potential conflict of interest.

## Publisher's Note

All claims expressed in this article are solely those of the authors and do not necessarily represent those of their affiliated organizations, or those of the publisher, the editors and the reviewers. Any product that may be evaluated in this article, or claim that may be made by its manufacturer, is not guaranteed or endorsed by the publisher.

## References

[1] Roth GA Johnson C Abajobir A Abd-Allah F Abera SF Abyu G. Global, Regional, and National Burden of Cardiovascular Diseases for 10 Causes, 1990 to (2015) J Am Coll Cardiol. (2017) 70. 10.1016/j.jacc.2017.04.052 28527533 PMC5491406

[2] Joseph P Leong D McKee M Anand SS Schwalm JD Teo K. Reducing the global burden of cardiovascular disease, part 1: the epidemiology and risk factors. Circulation Res. (2017) 121:677–94. 10.1161/CIRCRESAHA.117.308903 28860318

[3] Ruparelia N Chai JT Fisher EA Choudhury R. Inflammatory processes in cardiovascular disease: a route to targeted therapies. Nature reviews. Cardiology. (2017) 14:133–44. 10.1038/nrcardio.2016.185 27905474 PMC5525550

[4] Heinrich PC Behrmann I Haan S Hermanns HM Müller-Newen G Schaper F. Principles of interleukin (IL)-6-type cytokine signalling and its regulation. Biochemical J. (2003) 374. 10.1042/bj20030407 12773095 PMC1223585

[5] Unver N McAllister F. IL-6 family cytokines: Key inflammatory mediators as biomarkers and potential therapeutic targets. Cytokine Growth Factor Rev. (2018) 41:10–17. 10.1016/j.cytogfr.2018.04.004 29699936 PMC6085880

[6] Rose-John S. Interleukin-6 family cytokines. Cold Spring Harb Perspect Biol. (2018) 10. 10.1101/cshperspect.a028415 28620096 PMC5793756

[7] Jones SA Jenkins BJ. Recent insights into targeting the IL-6 cytokine family in inflammatory diseases and cancer. Nature reviews. Immunology. (2018) 18:773–89. 10.1038/s41577-018-0066-7 30254251

[8] Kang S Tanaka T Narazaki M Kishimoto T. Targeting interleukin-6 signaling in clinic. Immunity. (2019) 50:1007–23. 10.1016/j.immuni.2019.03.026 30995492

[9] Leonard H. Calabrese and Stefan Rose-John, *IL-6 biology: implications for clinical targeting in rheumatic disease*. Nature reviews Rheumatology. (2014) 10:720–7. 10.1038/nrrheum.2014.127 25136784

[10] Modares NF Polz R Haghighi F Lamertz L Behnke K Zhuang Y. IL-6 trans-signaling controls liver regeneration after partial hepatectomy. Hepatology (Baltimore, Md.). (2019) 70:2075–91. 10.1002/hep.30774 31100194

[11] Zegeye MM Lindkvist M Fälker K Kumawat AK Paramel G Grenegård M., Activation of the JAK/STAT3 and PI3K/AKT pathways are crucial for IL-6 trans-signaling-mediated pro-inflammatory response in human vascular endothelial cells Cell Commun Signal. (2018) 16:55. 10.1186/s12964-018-0268-4 30185178 PMC6125866

[12] Schaper F Rose-John S. Interleukin-6: Biology, signaling and strategies of blockade. Cytokine Growth Factor Rev. (2015) 26:475–487. 10.1016/j.cytogfr.2015.07.004 26189695

[13] Quintana FJ. Old dog, new tricks: IL-6 cluster signaling promotes pathogenic T17 cell differentiation. Nature Immunology. (2016) 18. 10.1038/ni.3637 27984572

[14] Mauer J Chaurasia B Goldau J Vogt MC Ruud J Nguyen KD. Signaling by IL-6 promotes alternative activation of macrophages to limit endotoxemia and obesity-associated resistance to insulin. Nature Immunology. (2014) 15:423–30. 10.1038/ni.2865 24681566 PMC4161471

[15] Braune J Weyer U Hobusch C Mauer J Brüning JC Ingo Bechmann. IL-6 regulates M2 polarization and local proliferation of adipose tissue macrophages in obesity. J Immunol. (Baltimore, Md.: 1950). (2017) 198:2927–34. 10.4049/jimmunol.1600476 28193830

[16] Ataie-Kachoie P Pourgholami MH Morris DL. Inhibition of the IL-6 signaling pathway: a strategy to combat chronic inflammatory diseases and cancer. Cytokine Growth Factor Rev. (2013) 24:163–173. 10.1016/j.cytogfr.2012.09.001 23107589

[17] Oberg HH Wesch D Grüssel S Rose-John S Kabelitz D. Differential expression of CD126 and CD130 mediates different STAT-3 phosphorylation in CD4+CD25- and CD25high regulatory T cells. Int Immunol. (2006) 18:555–63. 10.1093/intimm/dxh396 16540526

[18] Xu DH Zhu Z Wakefield MR Xiao H Bai Q Fang Y. The role of IL-11 in immunity and cancer. Cancer letters. (2016) 373:156–63. 10.1016/j.canlet.2016.01.004 26826523

[19] Koch L Kespohl B Agthe M Schumertl T Düsterhöft S Lemberg MK. Interleukin-11 (IL-11) receptor cleavage by the rhomboid protease RHBDL2 induces IL-11 trans-signaling. FASEB J. (2021) 35:e21380. 10.1096/fj.202002087R 33566379 PMC12266321

[20] Metcalfe RD Putoczki TL Griffin MDW. Structural understanding of interleukin 6 family cytokine signaling and targeted therapies: focus on interleukin 11. Front Immunol. (2020) 11:1424. 10.3389/fimmu.2020.01424 32765502 PMC7378365

[21] Liu X Wang Z Ye N Chen Z Zhou X Teng X. A protective role of IL-30 via STAT and ERK signaling pathways in macrophage-mediated inflammation. Biochem. Biophys. Res. Commun. (2013) 435:306–12. 10.1016/j.bbrc.2013.03.136 23583238

[22] Stumhofer JS Tait ED Quinn WJ Hosken N Spudy B Goenka R. A role for IL-27p28 as an antagonist of gp130-mediated signaling. Nature Immunology. (2010) 11:1119–26. 10.1038/ni.1957 21057510 PMC3059498

[23] Yan J Mitra A Hu J Cutrera JJ Xia X Doetschman T. Interleukin-30 (IL27p28) alleviates experimental sepsis by modulating cytokine profile in NKT cells. J Hepatology. (2016) 64:1128–36. 10.1016/j.jhep.2015.12.020 26767500 PMC4834232

[24] Kourko O Seaver K Odoardi N Basta S Gee K IL-27 IL-30 and IL-35: a cytokine triumvirate in cancer. Front Oncology. (2019) 9:969. 10.3389/fonc.2019.00969 31681561 PMC6797860

[25] Zhang Q Putheti P Zhou Q Liu Q Gao W Structures Structures and biological functions of IL-31 and IL-31 receptors. Cytokine Growth Factor Rev. (2008) 19:347–56. 10.1016/j.cytogfr.2008.08.003 18926762 PMC2659402

[26] Murdaca G Greco M Tonacci A Negrini S Borro M Puppo F. IL-33/IL-31 axis in immune-mediated and allergic diseases. Int J Mol Sci. (2019) 20. 10.3390/ijms20235856 31766607 PMC6929191

[27] Nakashima C Otsuka A Kabashima K. Interleukin-31 and interleukin-31 receptor: New therapeutic targets for atopic dermatitis. Experimental Dermatology. (2018) 27:327–31. 10.1111/exd.13533 29524262

[28] Hermanns HM. Oncostatin M and interleukin-31: Cytokines, receptors, signal transduction and physiology. Cytokine Growth Factor Rev. (2015) 26:545–58. 10.1016/j.cytogfr.2015.07.006 26198770

[29] Richards CD. The enigmatic cytokine oncostatin m and roles in disease. ISRN Inflammation. (2013) 2013:512103. 10.1155/2013/512103 24381786 PMC3870656

[30] Son H-S Lee SH Lee SY Kim EK Yang EJ Kim JK. Oncostatin M suppresses activation of IL-17/Th17 via SOCS3 regulation in CD4+ T cells. J Immunol (Baltimore, Md.: 1950). (2017) 198:1484–91. 10.4049/jimmunol.1502314 28093521 PMC5292586

[31] Jung ID Noh KT Lee CM Chun SH Jeong SK Park JW. Oncostatin M induces dendritic cell maturation and Th1 polarization. Biochem Biophys Res Commun. (2010) 394:272–8. 10.1016/j.bbrc.2010.02.153 20206608

[32] Nicola N Babon JJ. Leukemia inhibitory factor (LIF). Cytokine Growth Factor Rev. (2015) 26:533–44. 10.1016/j.cytogfr.2015.07.001 26187859 PMC4581962

[33] Metcalfe SM. LIF in the regulation of T-cell fate and as a potential therapeutic. Genes Immunity. (2011) 12:157–68. 10.1038/gene.2011.9 21368774

[34] Schuster B Kovaleva M Sun Y Regenhard P Matthews V Grötzinger J. Signaling of human ciliary neurotrophic factor (CNTF) revisited. The interleukin-6 receptor can serve as an alpha-receptor for CTNF. J Biological Chemistry. (2003) 278:9528–35. 10.1074/jbc.M210044200 12643274

[35] Fantone S Tossetta G Montironi R Senzacqua T Marzioni D Mazzucchelli R. Ciliary neurotrophic factor (CNTF) and its receptor (CNTFRα) signal through MAPK/ERK pathway in human prostate tissues: a morphological and biomolecular study. Eur J Histochemi: EJH. (2020) 64. 10.4081/ejh.2020.3147 33131268 PMC7586252

[36] Wen R Tao W Yiwen Li Paul A. Sieving, CNTF and retina. Progress in retinal and eye research. (2012) 31:136–151. 10.1016/j.preteyeres.2011.11.005 22182585 PMC3288688

[37] Ma B García-Cenador JM Lopez-Novoa J. Díez F. J. García-Criado Effects and mechanism of organ protection by cardiotrophin-1. Current medicinal chemistry. (2013) 20:246–256. 10.2174/092986713804806702 23244580

[38] Carneros D Santamaría EM Larequi E Vélez-Ortiz JM Reboredo M Mancheño U. Cardiotrophin-1 is an anti-inflammatory cytokine and promotes IL-4-induced M2 macrophage polarization. FASEB J. (2019) 33:7578–87. 10.1096/fj.201801563R 30892966

[39] Kuwahara K Saito Y Harada M Ishikawa M Ogawa E Miyamoto Y., Involvement of cardiotrophin-1 in cardiac myocyte-nonmyocyte interactions during hypertrophy of rat cardiac myocytes *in vitro*. Circulation. (1999) 100:1116–24. 10.1161/01.CIR.100.10.1116 10477538

[40] Stejskal D Ruzicka V. Cardiotrophin-1. Review. Biomedical papers of the Medical Faculty of the University Palacky, Olomouc. Czechoslovakia. (2008) 152. 10.5507/bp.2008.002 18795069

[41] Larsen JV Kristensen AM Pallesen LT Bauer J Vægter CB Nielsen MS. Cytokine-like factor 1, an essential facilitator of cardiotrophin-like cytokine:ciliary neurotrophic factor receptor α signaling and sorLA-Mediated turnover. Mol Cellular Biol. (2016) 36:1272–86. 10.1128/MCB.00917-15 26858303 PMC4836274

[42] Elson GC Lelièvre E Guillet C Chevalier S Plun-Favreau H Froger J. CLF associates with CLC to form a functional heteromeric ligand for the CNTF receptor complex. Nature Neuroscienc. (2000) 3:867–72. 10.1038/78765 10966616

[43] Kass DJ Yu G Loh KS Savir A Borczuk A Kahloon R. Cytokine-like factor 1 gene expression is enriched in idiopathic pulmonary fibrosis and drives the accumulation of CD4+ T cells in murine lungs: evidence for an antifibrotic role in bleomycin injury. Am J Pathol. (2012) 180:1963–78. 10.1016/j.ajpath.2012.01.010 22429962 PMC3354590

[44] Pasquin S Laplante V Kouadri S Milasan A Mayer G Tormo AJ. Cardiotrophin-like cytokine increases macrophage-foam cell transition. J Immunology (Baltimore, Md.: 1950). (2018) 201:2462–71. 10.4049/jimmunol.1800733 30209193

[45] Pasquin S Tormo A Moreau J Laplante V Sharma M Gauchat JF. Cardiotrophin-like cytokine factor 1 exhibits a myeloid-biased hematopoietic-stimulating function. Front Immunology. (2019) 10:2133. 10.3389/fimmu.2019.02133 31552057 PMC6746841

[46] Vlotides G Zitzmann K Stalla GK Auernhammer CJ. Novel neurotrophin-1/B cell-stimulating factor-3 (NNT-1/BSF-3)/cardiotrophin-like cytokine (CLC)–a novel gp130 cytokine with pleiotropic functions. Cytokine Growth Factor Rev. (2004) 15:325–36. 10.1016/j.cytogfr.2004.04.002 15450249

[47] Cui Y Dai W Li Y Circulating Circulating levels of sgp130 and sex hormones in male patients with coronary atherosclerotic disease. Atherosclerosis. (2017) 266:151–7. 10.1016/j.atherosclerosis.2017.09.002 29028483

[48] Dong C. TH17 cells in development: an updated view of their molecular identity and genetic programming. Nature reviews. Immunology. (2008) 8:337–48. 10.1038/nri2295 18408735

[49] Tosolini M Kirilovsky A Mlecnik B Fredriksen T Mauger S Bindea G. Clinical impact of different classes of infiltrating T cytotoxic and helper cells (Th1, th2, treg, th17) in patients with colorectal cancer. Cancer Res. (2011) 71:1263–71. 10.1158/0008-5472.CAN-10-2907 21303976

[50] Yin Z Ma T Lin Y Lu X Zhang C Chen S. IL-6/STAT3 pathway intermediates M1/M2 macrophage polarization during the development of hepatocellular carcinoma. J Cell Biochem. (2018) 119:9419–32. 10.1002/jcb.27259 30015355

[51] Prabhu SD Frangogiannis NG. The biological basis for cardiac repair after myocardial infarction: from inflammation to fibrosis. Circulation Res. (2016) 119. 10.1161/CIRCRESAHA.116.303577 27340270 PMC4922528

[52] Del Pinto R Ferri C. Inflammation-accelerated senescence and the cardiovascular system: mechanisms and perspectives. Int J Mol Sci. (2018) 19. 10.3390/ijms19123701 30469478 PMC6321367

[53] Ruparelia N Choudhury R. Inflammation and atherosclerosis: what is on the horizon? Heart (British Cardiac Society). (2020) 106:80–5. 10.1136/heartjnl-2018-314230 31843811

[54] Guzik TJ Touyz RM. Oxidative stress, inflammation, and vascular aging in hypertension. Hypertension. (2017) 70:660–67. 10.1161/HYPERTENSIONAHA.117.07802 28784646

[55] Murakami M Kamimura D Hirano T. Pleiotropy and specificity: insights from the interleukin 6 family of cytokines. Immunity. (2019) 50:812–31. 10.1016/j.immuni.2019.03.027 30995501

[56] Kang S Narazaki M. Hozaifa Metwally, and Tadamitsu Kishimoto, Historical overview of the interleukin-6 family cytokine. J Exp Med. (2020) 217. 10.1084/jem.20190347 32267936 PMC7201933

[57] Erling Falk Pathogenesis Pathogenesis of atherosclerosis. J Am Coll Cardiol. (2006) 47:C7–12. 10.1016/j.jacc.2005.09.068 16631513

[58] Ross R. Atherosclerosis–an inflammatory disease. N Engl J Med. (1999) 340:115–26. 10.1056/NEJM199901143400207 9887164

[59] Okazaki S Sakaguchi M Miwa K Furukado S Yamagami H Yagita Y. Association of interleukin-6 with the progression of carotid atherosclerosis: a 9-year follow-up study. Stroke. (2014) 45:2924–9. 10.1161/STROKEAHA.114.005991 25139874

[60] Hartman J Frishman WH Inflammation Inflammation and atherosclerosis: a review of the role of interleukin-6 in the development of atherosclerosis and the potential for targeted drug therapy. Cardiology Rev. (2014) 22:147–51. 10.1097/CRD.0000000000000021 24618929

[61] Schuett H Luchtefeld M Grothusen C Grote K Schieffer B. How much is too much? Interleukin-6 and its signalling in atherosclerosis. Thrombosis and Haemostasis. (2009) 102:215–22. 10.1160/TH09-05-0297 19652871

[62] Wassmann S Stumpf M Strehlow K Schmid A Schieffer B Böhm M. Interleukin-6 induces oxidative stress and endothelial dysfunction by overexpression of the angiotensin II type 1 receptor. Circulation Res. (2004) 94:534–41. 10.1161/01.RES.0000115557.25127.8D 14699015

[63] Groot HE Ali LA Iwan C Horst V Schurer RAJ van der Werf HW., Plasma interleukin 6 levels are associated with cardiac function after ST-elevation myocardial infarction. Clin Res Cardiol. (2019) 108:612–21. 10.1007/s00392-018-1387-z 30367209 PMC6529378

[64] Lindmark E Diderholm E Wallentin L Siegbahn A. Relationship between interleukin 6 and mortality in patients with unstable coronary artery disease: effects of an early invasive or noninvasive strategy. JAMA. (2001) 286:2107–13. 10.1001/jama.286.17.2107 11694151

[65] Fisman EZ Benderly M Esper RJ Behar S Boyko V Adler Y., Interleukin-6 and the risk of future cardiovascular events in patients with angina pectoris and/or healed myocardial infarction. Am J Cardiology. (2006) 98:14–18. 10.1016/j.amjcard.2006.01.045 16784912

[66] Kleveland O Kunszt G Bratlie M Ueland T Broch K Holte E., Effect of a single dose of the interleukin-6 receptor antagonist tocilizumab on inflammation and troponin T release in patients with non-ST-elevation myocardial infarction: a double-blind, randomized, placebo-controlled phase 2 trial. Eur Heart J. (2016) 37:2406–13. 10.1093/eurheartj/ehw171 27161611

[67] Ridker PM Libby P MacFadyen JG Thuren T Ballantyne C Fonseca F. Modulation of the interleukin-6 signalling pathway and incidence rates of atherosclerotic events and all-cause mortality: analyses from the Canakinumab Anti-Inflammatory Thrombosis Outcomes Study (CANTOS) Eur Heart J. (2018) 39:3499–507. 10.1093/eurheartj/ehy310 30165610

[68] Huber SA Conze SD Hardin N Tracy R. Interleukin-6 exacerbates early atherosclerosis in mice. Arterioscler Thromb Vasc. (1999) 19:2364–7. 10.1161/01.ATV.19.10.2364 10521365

[69] Schieffer B Selle T Hilfiker A Hilfiker-Kleiner D Grote K Tietge UJF. Impact of interleukin-6 on plaque development and morphology in experimental atherosclerosis. Circulation. (2004) 110:3493–500. 10.1161/01.CIR.0000148135.08582.97 15557373

[70] Madan M Bishayi B Hoge M Amar S. Atheroprotective role of interleukin-6 in diet- and/or pathogen-associated atherosclerosis using an ApoE heterozygote murine model. Atherosclerosis. (2008) 197:504–14. 10.1016/j.atherosclerosis.2007.02.023 17412346 PMC2430020

[71] Albasanz-Puig A Murray J Preusch M Coan D Namekata M Patel Y. Oncostatin M is expressed in atherosclerotic lesions: a role for Oncostatin M in the pathogenesis of atherosclerosis. Atherosclerosis. (2011) 216:292–8. 10.1016/j.atherosclerosis.2011.02.003 21376322

[72] Zhang X Li J Qin J Cheng WL Zhu X Gong FH. Oncostatin M receptor β deficiency attenuates atherogenesis by inhibiting JAK2/STAT3 signaling in macrophages. J Lipid Res. (2017) 58:895–906. 10.1194/jlr.M074112 28258089 PMC5408608

[73] Li X Zhang X Wei L Xia Y Guo X. Relationship between serum oncostatin M levels and degree of coronary stenosis in patients with coronary artery disease. Clinical Lab. (2014) 60:113–8. 10.7754/Clin.Lab.2013.121245 24600984

[74] van Keulen D Pouwer MG Emilsson V Matic LP Pieterman EJ Hedin U. Oncostatin M reduces atherosclerosis development in APOE^*^3Leiden.CETP mice and is associated with increased survival probability in humans. PloS ONE. (2019) 14:e0221477. 10.1371/journal.pone.0221477 31461490 PMC6713386

[75] Rolfe B Stamatiou S World CJ Brown L Thomas AC Bingley JA. Leukaemia inhibitory factor retards the progression of atherosclerosis. Cardiovascular Res. (2003) 58:222–230. 10.1016/S0008-6363(02)00832-5 12667965

[76] Moran CS Campbell JH Simmons DL Campbell GR Human Human leukemia inhibitory factor inhibits development of experimental atherosclerosis. Arterioscler Thromb Vasc Biol. (1994) 14:1356–163. 10.1161/01.ATV.14.8.1356 8049198

[77] Konii H Sato K Kikuchi S Okiyama H Watanabe R Hasegawa A. Stimulatory effects of cardiotrophin 1 on atherosclerosis. Hypertension (Dallas, Tex.: 1979). (2013) 62:942–50. 10.1161/HYPERTENSIONAHA.113.01653 24041953

[78] Miteva K Baptista D Montecucco F Asrih M Burger F Roth A. Cardiotrophin-1 deficiency abrogates atherosclerosis progression. Scientific Reports. (2020) 10:5791. 10.1038/s41598-020-62596-6 32238841 PMC7113288

[79] Suzuki H Kurihara Y Takeya M Kamada N Kataoka M Jishage K., A role for macrophage scavenger receptors in atherosclerosis and susceptibility to infection. Nature. (1997) 386:292–6. 10.1038/386292a0 9069289

[80] Kim JW Marquez C Sperberg RAP Wu J Bae WG Huang PS. Engineering a potent receptor superagonist or antagonist from a novel IL-6 family cytokine ligand. Proc Natl Acad Sci U. S. A. (2020) 117:14110–18. 10.1073/pnas.1922729117 32522868 PMC7322068

[81] Drummond GR Vinh A Guzik TJ Sobey CG. Immune mechanisms of hypertension. Nature reviews. Immunology. (2019) 19:517–32. 10.1038/s41577-019-0160-5 30992524

[82] Hashmat S Rudemiller N Lund H Abais-Battad JM Why SV Mattson DL. Interleukin-6 inhibition attenuates hypertension and associated renal damage in Dahl salt-sensitive rats. Am J Physiol Renal Physiol. (2016) 311:F555–61. 10.1152/ajprenal.00594.2015 27279492 PMC5142167

[83] Lee DL Sturgis LC Labazi H Osborne JB Fleming C Pollock JS. Angiotensin II hypertension is attenuated in interleukin-6 knockout mice. American journal of physiology. Heart and Circulatory Physiol. (2006) 290:H935–H940. 10.1152/ajpheart.00708.2005 16284237

[84] Brands MW Banes-Berceli AKL Inscho EW Al-Azawi H Allen AJ Labazi H. Interleukin 6 knockout prevents angiotensin II hypertension: role of renal vasoconstriction and janus kinase 2/signal transducer and activator of transcription 3 activation. Hypertension (Dallas, Tex.: 1979). (2010) 56:879–84. 10.1161/HYPERTENSIONAHA.110.158071 20921429 PMC3500610

[85] Chae CU Lee RT Rifai N Ridker M. Blood pressure and inflammation in apparently healthy men. Hypertension (Dallas, Tex.: 1979). (2001) 38:399–403. 10.1161/01.HYP.38.3.399 11566912

[86] Mao SQ Sun JH Gu JL Zhu FB Yin FY Zhang LN. Hypomethylation of interleukin-6 (IL-6) gene increases the risk of essential hypertension: a matched case-control study. J Human Hypertension. (2017) 31:530–6. 10.1038/jhh.2017.7 28300071

[87] Gkaliagkousi E Gavriilaki E Nikolaidou B Chatzopoulou B Anyfanti P Triantafyllou A., Association between cardiotrophin 1 levels and central blood pressure in untreated patients with essential hypertension. Am J Hypertension. (2014) 27:651–5. 10.1093/ajh/hpt238 24401751

[88] Pemberton CJ Raudsepp SD Yandle TG Cameron VA Richards AM. Plasma cardiotrophin-1 is elevated in human hypertension and stimulated by ventricular stretch. Cardiovascular Res. (2005) 68:109–17. 10.1016/j.cardiores.2005.05.014 15978561

[89] López B Castellano JM González A Barba J Díez J. Association of increased plasma cardiotrophin-1 with inappropriate left ventricular mass in essential hypertension. Hypertension (Dallas, Tex.: 1979). (2007) 50:977–83. 10.1161/HYPERTENSIONAHA.107.098111 17846346

[90] Mahmud A Feely J. Arterial stiffness is related to systemic inflammation in essential hypertension. Hypertension (Dallas, Tex.: 1979). (2005) 46:1118–22. 10.1161/01.HYP.0000185463.27209.b0 16216991

[91] Tuttolomondo A Pecoraro R Buttà C Di Raimondo D Ferrante A Della Corte V. Arterial stiffness indexes and serum cytokine levels in seronegative spondyloarthritis: relationships between stiffness markers and metabolic and immunoinflammatory variables. Scand J Rheumatol. (2015) 44:474–479. 10.3109/03009742.2015.1030449 26169842

[92] Tuttolomondo A Di Raimondo D Pecoraro R Serio A D'Aguanno G Pinto A. Immune-inflammatory markers and arterial stiffness indexes in subjects with acute ischemic stroke. Atherosclerosis. (2010) 213:311–8. 10.1016/j.atherosclerosis.2010.08.065 20889155

[93] Desjardins MP Sidibé A Fortier C Mac-Way F Marquis K De Serres S. Association of interleukin-6 with aortic stiffness in end-stage renal disease. J Am Soc Hypertension: JASH. (2018) 12. 10.1016/j.jash.2017.09.013 29170076

[94] Du B Ouyang A Eng JS Fleenor BS. Aortic perivascular adipose-derived interleukin-6 contributes to arterial stiffness in low-density lipoprotein receptor deficient mice. American journal of physiology. Heart Circulat Physiol. (2015) 308:H1382–H1390. 10.1152/ajpheart.00712.2014 25840831 PMC4451307

[95] Johnston KW Rutherford RB Tilson MD Shah DM Hollier L Stanley JC. Suggested standards for reporting on arterial aneurysms. Subcommittee on Reporting Standards for Arterial Aneurysms, Ad Hoc Committee on Reporting Standards, Society for Vascular Surgery and North American Chapter, International Society for Cardiovascular Surgery. J Vascular Surgery. (1991) 13:452–8. 10.1067/mva.1991.26737 1999868

[96] Guo DC Papke CL He R Milewicz DM. Pathogenesis of thoracic and abdominal aortic aneurysms. Ann N Y Acad Sci. (2006) 1085:339–52. 10.1196/annals.1383.013 17182954

[97] Silaschi M Byrne J Wendler O. Aortic dissection: medical, interventional and surgical management. Heart (British Cardiac Society). (2017) 103:78–87. 10.1136/heartjnl-2015-308284 27733536

[98] Cai YL Wang ZW The The expression and significance of IL-6 IFN-γ SM22α and and MMP-2 in rat model of aortic dissection. Eur Rev Med Pharmacol Sci. (2017) 21:560–8. 28239811

[99] Dawson J Cockerill GW Choke E Belli AM Loftus I Thompson MM. Aortic aneurysms secrete interleukin-6 into the circulation. J Vasc Surg Cases. (2007) 45:350–6. 10.1016/j.jvs.2006.09.049 17264016

[100] Dawson J Cockerill G Choke E Loftus I Thompson MM. Aortic aneurysms as a source of circulating interleukin-6. Ann N Y Acad Sci. (2006) 1085:320–323. 10.1196/annals.1383.009 17182950

[101] Paige E Clément M Lareyre F Sweeting M Raffort J Grenier C. Interleukin-6 receptor signaling and abdominal aortic aneurysm growth rates. Circulation Genomic Precision Med. (2019) 12:e002413. 10.1161/CIRCGEN.118.002413 30657332 PMC6383754

[102] Lv XC Lin Y Wu QS Wang L Hou YT Dong Y. Plasma interleukin-6 is a potential predictive biomarker for postoperative delirium among acute type a aortic dissection patients treated with open surgical repair. J Cardiothoracic Surg. (2021) 16:146. 10.1186/s13019-021-01529-4 34044881 PMC8161913

[103] Wu Q Li J Chen L Yan LL Qiu Z Shen Y. Efficacy of interleukin-6 in combination with D-dimer in predicting early poor postoperative prognosis after acute stanford type a aortic dissection. J Cardiothoracic Surg. (2020) 15:172. 10.1186/s13019-020-01206-y 32677975 PMC7364558

[104] Xu Y Ye J Wang M Wang Y Ji Q Huang Y. Increased interleukin-11 levels in thoracic aorta and plasma from patients with acute thoracic aortic dissection. Clinica Chimica Acta. (2018) 481:193–9. 10.1016/j.cca.2018.03.014 29555322

[105] Middleton RK Lloyd GM Bown MJ Cooper NJ London NJ Sayers RD. The pro-inflammatory and chemotactic cytokine microenvironment of the abdominal aortic aneurysm wall: a protein array study. J Vasc Surg Cases. (2007) 45:574–80. 10.1016/j.jvs.2006.11.020 17321344

[106] Modur V Feldhaus MJ Weyrich AS Jicha DL Prescott SM Zimmerman GA. Oncostatin M is a proinflammatory mediator. In vivo effects correlate with endothelial cell expression of inflammatory cytokines and adhesion molecules. J Clin Investig. (1997) 100:158–68. 10.1172/JCI119508 9202068 PMC508176

[107] Tokito A Jougasaki M Ichiki T Hamasaki S. Cardiotrophin-1 induces matrix metalloproteinase-1 in human aortic endothelial cells. PloS ONE. (2013) 8:e68801. 10.1371/journal.pone.0068801 23935888 PMC3720803

[108] Li Y Yang D Sun B Zhang X Li X Liu Z. Discovery of crucial cytokines associated with abdominal aortic aneurysm formation by protein array analysis. Exp Biol Med. (2019) 244:1648–57. 10.1177/1535370219885101 31665916 PMC6963380

[109] Frangogiannis NG. Cardiac fibrosis: Cell biological mechanisms, molecular pathways and therapeutic opportunities. Mol Aspects Med. (2019) 65:70–99. 10.1016/j.mam.2018.07.001 30056242

[110] Bacmeister L Schwarzl M Warnke S Stoffers B Blankenberg S Westermann D. Inflammation and fibrosis in murine models of heart failure. Basic Research In Cardiology. (2019) 114:19. 10.1007/s00395-019-0722-5 30887214

[111] Charles J Pollack A Miller G. Cardiomyopathy. Australian Family Physician. (2014) 43:253. 24791762

[112] Yancy CW Jessup M Bozkurt B Butler J Casey DE Drazner H. 2013 ACCF/AHA guideline for the management of heart failure: a report of the American College of Cardiology Foundation/American Heart Association Task Force on Practice Guidelines. J Am Coll Cardiol. (2013) 62:e147–e239. 23747642 10.1016/j.jacc.2013.05.019

[113] Eskandari V Amirzargar AA Mahmoudi MJ Rahnemoon Z Rahmani F Sadati S. Gene expression and levels of IL-6 and TNFα in PBMCs correlate with severity and functional class in patients with chronic heart failure. Irish J Med Sci. (2018) 187:359–68. 10.1007/s11845-017-1680-2 28889349

[114] Kumar S Wang G Zheng N Cheng W Ouyang K Lin H. HIMF (Hypoxia-Induced Mitogenic Factor)-IL (Interleukin)-6 Signaling mediates cardiomyocyte-fibroblast crosstalk to promote cardiac hypertrophy and fibrosis. Hypertension (Dallas, Tex.: 1979). (2019) 73:1058–70. 10.1161/HYPERTENSIONAHA.118.12267 30827145

[115] Wang JH Zhao L Pan X Chen NN Chen J Gong QL., Hypoxia-stimulated cardiac fibroblast production of IL-6 promotes myocardial fibrosis via the TGF-β1 signaling pathway. Lab Invest. (2016) 96:839–52. 10.1038/labinvest.2016.65 27348628

[116] Jing R Long TY Pan W Li F Xie QY. IL-6 knockout ameliorates myocardial remodeling after myocardial infarction by regulating activation of M2 macrophages and fibroblast cells. Eur Rev Med Pharmacol Sci. (2019) 23:6283–6291. 31364133 10.26355/eurrev_201907_18450

[117] Schafer S Viswanathan S Widjaja AA Lim WW Moreno-Moral A DeLaughter DM. IL-11 is a crucial determinant of cardiovascular fibrosis. Nature. (2017) 552:110–115. 10.1038/nature24676 29160304 PMC5807082

[118] Corden B Lim WW Song W Chen X Ko NSJ Su L. Therapeutic targeting of interleukin-11 signalling reduces pressure overload-induced cardiac fibrosis in mice. J Cardiovasc Transl Res. (2020) 14:222–8. 10.1007/s12265-020-10054-z 32592090

[119] Obana M Maeda M Takeda K Hayama A Mohri T Yamashita T. Therapeutic activation of signal transducer and activator of transcription 3 by interleukin-11 ameliorates cardiac fibrosis after myocardial infarction. Circulation. (2010) 121:684–691. 10.1161/CIRCULATIONAHA.109.893677 20100971

[120] Abe H Takeda N Isagawa T Semba H Nishimura S Suimye Morioka. Macrophage hypoxia signaling regulates cardiac fibrosis via Oncostatin M. Nature Communications. (2019) 10:2824. 10.1038/s41467-019-10859-w 31249305 PMC6597788

[121] Zou Y Takano H Mizukami M Akazawa H Qin J Toko H. Leukemia inhibitory factor enhances survival of cardiomyocytes and induces regeneration of myocardium after myocardial infarction. Circulation. (2003) 108:748–53. 10.1161/01.CIR.0000081773.76337.44 12860906

[122] Zgheib C Zouein FA Kurdi M Booz GW. Chronic treatment of mice with leukemia inhibitory factor does not cause adverse cardiac remodeling but improves heart function. European Cytokine Network. (2012) 23:191–197. 10.1684/ecn.2012.0319 23291613 PMC3595094

[123] López B González A Querejeta R Larman M Rábago G Díez J. Association of cardiotrophin-1 with myocardial fibrosis in hypertensive patients with heart failure. Hypertension (Dallas, Tex.: 1979). (2014) 63:483–9. 10.1161/HYPERTENSIONAHA.113.02654 24366078

[124] Martínez-Martínez E Brugnolaro C Ibarrola J Ravassa S Buonafine M López B. CT-1 (Cardiotrophin-1)-Gal-3 (Galectin-3) Axis in cardiac fibrosis and inflammation. Hypertension (Dallas, Tex.: 1979). (2019) 73:602–11. 10.1161/HYPERTENSIONAHA.118.11874 30612490

[125] Roig E Orús J Paré C Azqueta M Filella X Perez-Villa F. Serum interleukin-6 in congestive heart failure secondary to idiopathic dilated cardiomyopathy. Am J Cardiology. (1998) 82. 10.1016/S0002-9149(98)00388-9 9732906

[126] Scally C Abbas H Ahearn T Srinivasan J Mezincescu A Rudd A. Myocardial and systemic inflammation in acute stress-induced (takotsubo) cardiomyopathy. Circulation. (2019) 139:1581–92. 10.1161/CIRCULATIONAHA.118.037975 30586731 PMC6438459

[127] Li Q Ye WX Huang ZJ Zhang Q He YF. Effect of IL-6-mediated STAT3 signaling pathway on myocardial apoptosis in mice with dilated cardiomyopathy. Eur Rev Med Pharmacol Sci. (2019) 23:3042–3050. 31002169 10.26355/eurrev_201904_17586

[128] Zhang E Ma S Zhang R Li S Zhu D Han D. Oncostatin M-induced cardiomyocyte dedifferentiation regulates the progression of diabetic cardiomyopathy through B-Raf/Mek/Erk signaling pathway. Acta biochimica et biophysica Sinica. (2016) 48:257–65. 10.1093/abbs/gmv137 26837420 PMC4885130

[129] Kubin T Pöling J Kostin S Gajawada P Hein S Rees W. Oncostatin M is a major mediator of cardiomyocyte dedifferentiation and remodeling. Cell Stem Cell. (2011) 9:420–32. 10.1016/j.stem.2011.08.013 22056139

[130] Monserrat L López B González A Hermida M Fernández X Ortiz M. Cardiotrophin-1 plasma levels are associated with the severity of hypertrophy in hypertrophic cardiomyopathy. Eur Heart J. (2011) 32:177–83. 10.1093/eurheartj/ehq400 21059734 PMC3021387

[131] Chandrasekar B Melby C Pennica D Freeman GL. Overexpression of cardiotrophin-1 and gp130 during experimental acute Chagasic cardiomyopathy. Immunology Letters. (1998) 61:89–95. 10.1016/S0165-2478(97)00167-3 9657259

[132] Tsutamoto T Wada A Maeda K Mabuchi N Hayashi M Tsutsui T. Relationship between plasma level of cardiotrophin-1 and left ventricular mass index in patients with dilated cardiomyopathy. J Am Coll Cardiol. (2001) 38:1485–90. 10.1016/S0735-1097(01)01576-5 11691527

[133] Markousis-Mavrogenis G Tromp J Ouwerkerk W Devalaraja M Anker SD Cleland JG. The clinical significance of interleukin-6 in heart failure: results from the BIOSTAT-CHF study. Eur J Heart Failure. (2019) 21:965–73. 10.1002/ejhf.1482 31087601

[134] Tsutamoto T Hisanaga T Wada A Maeda K Ohnishi M Fukai D., Interleukin-6 spillover in the peripheral circulation increases with the severity of heart failure, and the high plasma level of interleukin-6 is an important prognostic predictor in patients with congestive heart failure. J Am Coll Cardiol. (1998) 31:391–8. 10.1016/S0735-1097(97)00494-4 9462584

[135] Zhao L Cheng G Jin R Afzal MR Samanta A Xuan YT. Deletion of interleukin-6 attenuates pressure overload-induced left ventricular hypertrophy and dysfunction. Circulation Res. (2016) 118:1918–29. 10.1161/CIRCRESAHA.116.308688 27126808 PMC4902783

[136] Huo S Shi S Ma H Yan D Luo Guo J. Alleviation of inflammation and oxidative stress in pressure overload-induced cardiac remodeling and heart failure via IL-6/STAT3 inhibition by raloxifene. Oxid Med Cell Longev. (2021) 2021:6699054. 10.1155/2021/6699054 33824698 PMC8007383

[137] Ye Y Wang Z Ye D Wang Y Wang M Ji Q. Increased interleukin-11 levels are correlated with cardiac events in patients with chronic heart failure. Mediators Inflamm. (2019) 2019:1575410. 10.1155/2019/1575410 30728748 PMC6341241

[138] Gruson D Ferracin B Ahn SA Rousseau MF. Elevation of plasma oncostatin M in heart failure. Future Cardiology. (2017) 13:219–227. 10.2217/fca-2016-0063 28585906

[139] Hirota H Izumi M Hamaguchi T Sugiyama S Murakami E Kunisada K., Circulating interleukin-6 family cytokines and their receptors in patients with congestive heart failure. Heart and vessels. (2004) 19:237–241. 10.1007/s00380-004-0770-z 15372299

[140] Eiken HG Øie E Damås JK Yndestad A Bjerkeli V Aass H., Myocardial gene expression of leukaemia inhibitory factor, interleukin-6 and glycoprotein 130 in end-stage human heart failure. Eur J Clinical Investigation. (2001) 31:389–97. 10.1046/j.1365-2362.2001.00795.x 11380590

[141] Kanazawa H Ieda M Kimura K Arai T Kawaguchi-Manabe H Matsuhashi T. Heart failure causes cholinergic transdifferentiation of cardiac sympathetic nerves via gp130-signaling cytokines in rodents. J Clinical Investigation. (2010) 120:408–21. 10.1172/JCI39778 20051627 PMC2810079

[142] Celik A Sahin S Koc F Karayakali M Sahin M Benli I., aCardiotrophin-1 plasma levels are increased in patients with diastolic heart failure. Medical Science Monitor. (2012) 18:CR25-CR31. 10.12659/MSM.882197 22207116 PMC3560678

[143] Song K Wang S Huang B Luciano A Srivastava R Mani A. Plasma cardiotrophin-1 levels are associated with hypertensive heart disease: a meta-analysis. J Clinical Hypertension (Greenwich, Conn.). (2014) 16:686–92. 10.1111/jch.12376 25052897 PMC4159421

[144] Tsutamoto T Asai S Tanaka T Sakai H Nishiyama K Fujii M. Plasma level of cardiotrophin-1 as a prognostic predictor in patients with chronic heart failure. Eur J Heart Failure. (2007) 9:1032–37. 10.1016/j.ejheart.2007.07.015 17766177

[145] López N Varo N Díez J Antonia Fortuño M. Loss of myocardial LIF receptor in experimental heart failure reduces cardiotrophin-1 cytoprotection. A role for neurohumoral agonists? Cardiovascular Res. (2007) 75:536–545. 10.1016/j.cardiores.2007.04.025 17559824

[146] Yu-Feng Hu Yi-Jen Chen Yenn-Jiang Lin Shih-Ann Chen Inflammation Inflammation and the pathogenesis of atrial fibrillation. Nature reviews. Cardiology. (2015) 12:230–43. 10.1038/nrcardio.2015.2 25622848

[147] Savelieva I Kakouros N Kourliouros A Camm AJ. Upstream therapies for management of atrial fibrillation: review of clinical evidence and implications for European Society of Cardiology guidelines. Part II: secondary prevention. Europace: European pacing, arrhythmias, and cardiac electrophysiology: journal of the working groups on cardiac pacing, arrhythmias, and cardiac cellular electrophysiology of the European Society of Cardiology. (2011) 13:610–625. 10.1093/europace/eur023 21515595

[148] Amdur RL Mukherjee M Go A Barrows IR Ramezani A Shoji J. Interleukin-6 Is a Risk Factor for Atrial Fibrillation in Chronic Kidney Disease: Findings from the CRIC Study. PloS ONE. (2016) 11:e0148189. 10.1371/journal.pone.0148189 26840403 PMC4739587

[149] Marcus GM Whooley MA Glidden DV Pawlikowska L Zaroff JG Olgin JE. Interleukin-6 and atrial fibrillation in patients with coronary artery disease: data from the Heart and Soul Study. Am Heart Journal. (2008) 155:303–9. 10.1016/j.ahj.2007.09.006 18215601 PMC2247366

[150] Xu J Ren JF Mugelli A Belardinelli L Keith JC Jr Pelleg A. Age-dependent atrial remodeling induced by recombinant human interleukin-11: implications for atrial flutter/fibrillation. J Cardiovascular Pharmacology. (2002) 39:435–40. 10.1097/00005344-200203000-00015 11862123

[151] Xie J Zhu S Dai Q Lu J Chen J Li G. Oncostatin M was associated with thrombosis in patients with atrial fibrillation. Medicine. (2017) 96:e6806. 10.1097/MD.0000000000006806 28471981 PMC5419927

[152] Altun I Pamukcu B Yildiz CE Arkaya SC Guz G Yilmaz A. Cardiotrophin-1: A new predictor of atrial fibrillation relapses after successful cardioversion. Bosnian J Basic Medical Sciences. (2015) 15:68–73. 10.17305/bjbms.2015.503 26295297 PMC4593931

[153] Blauwet LA Cooper LT. Myocarditis. Prog Cardiovascular Dis. (2010) 52:274–88. 10.1016/j.pcad.2009.11.006 20109598 PMC5951175

[154] Sagar S Liu P Cooper LT. Myocarditis. Lancet (London, England). (2012) 379:738–47. 10.1016/S0140-6736(11)60648-X 22185868 PMC5814111

[155] Eriksson U Kurrer MO Schmitz N Marsch SC Fontana A Eugster HP., Interleukin-6-deficient mice resist development of autoimmune myocarditis associated with impaired upregulation of complement C3. Circulation. (2003) 107:320–325. 10.1161/01.CIR.0000043802.38699.66 12538435

[156] Yamashita T Iwakura T Matsui K Kawaguchi H Obana M Hayama A. IL-6-mediated Th17 differentiation through RORγt is essential for the initiation of experimental autoimmune myocarditis. Cardiovascular Res. (2011) 91:640–8. 10.1093/cvr/cvr148 21622681

[157] Tanaka T Kanda T McManus BM Kanai H Akiyama H Sekiguchi K. Overexpression of interleukin-6 aggravates viral myocarditis: impaired increase in tumor necrosis factor-alpha. J Molecular Cellular Cardiology. (2001) 33:1627–35. 10.1006/jmcc.2001.1428 11549342

[158] Okuno M Nakagawa M Shimada M Saito M Hishinuma S Takihara K. Expressional patterns of cytokines in a murine model of acute myocarditis: early expression of cardiotrophin-1. Lab Invest. (2000) 80:433–40. 10.1038/labinvest.3780048 10744079

[159] Hausenloy DJ Yellon DM. Myocardial ischemia-reperfusion injury: a neglected therapeutic target. J Clinical Investigation. (2013) 123. 10.1172/JCI62874 23281415 PMC3533275

[160] Boros P Bromberg JS New cellular and molecular immune pathways in ischemia/reperfusion injury. Am J Transplantation. (2006) 6:652–8. 10.1111/j.1600-6143.2005.01228.x 16539620

[161] Arslan F Kleijn D Pasterkamp G. Innate immune signaling in cardiac ischemia. Nature reviews. Cardiology. (2011) 8:292–300. 10.1038/nrcardio.2011.38 21448140

[162] Jong WC Cate HT Linnenbank AC de Boer OJ Reitsma PH de Winter RJ. Reduced acute myocardial ischemia-reperfusion injury in IL-6-deficient mice employing a closed-chest model. Inflammation Res. (2016) 65:489–499. 10.1007/s00011-016-0931-4 26935770 PMC4841857

[163] Obana M Miyamoto K Murasawa S Iwakura T Hayama A Yamashita T. Therapeutic administration of IL-11 exhibits the postconditioning effects against ischemia-reperfusion injury via STAT3 in the heart. Am J physiology. (2012) 303:H569–H577. 10.1152/ajpheart.00060.2012 22707562

[164] Tamura Y Kohno H Mohri T Fujio Y Matsumiya G. The cardioprotective effect of interleukin-11 against ischemia-reperfusion injury in a heart donor model. Annals Cardiothoracic Surg. (2018) 7 10.21037/acs.2017.09.11 29492387 PMC5827124

[165] Gwechenberger MM Moertl D Pacher R Huelsmann M. Oncostatin-M in myocardial ischemia/reperfusion injury may regulate tissue repair. Croatian Medical J. (2004) 45:149–57. 15103750

[166] Sun D Li S Wu S Zhang M Zhang X Wei L., Oncostatin M (OSM) protects against cardiac ischaemia/reperfusion injury in diabetic mice by regulating apoptosis, mitochondrial biogenesis and insulin sensitivity. J Cellular Mol Med. (2015) 19:1296–307. 10.1111/jcmm.12501 25752217 PMC4459845

[167] Zhang M Wang C Hu J Lin J Zhao Z Shen Z., Notch3/Akt signaling contributes to OSM-induced protection against cardiac ischemia/reperfusion injury. Apoptosis. (2015) 20:1150–63. 10.1007/s10495-015-1148-7 26093524

[168] Nelson SK Wong GH McCord JM. Leukemia inhibitory factor and tumor necrosis factor induce manganese superoxide dismutase and protect rabbit hearts from reperfusion injury. J Mol Cellular Cardiology. (1995) 27:223–9. 10.1016/S0022-2828(08)80021-1 7760346

[169] Liao Z Brar BK Cai Q Stephanou A O'Leary RH Pennica D. Cardiotrophin-1 (CT-1) can protect the adult heart from injury when added both prior to ischaemia and at reperfusion. Cardiovascular Res. (2002) 53:902–10. 10.1016/S0008-6363(01)00531-4 11922900

[170] Brar BK Stephanou A Liao ZO'Leary RM Pennica D Yellon DM., Cardiotrophin-1 can protect cardiac myocytes from injury when added both prior to simulated ischaemia and at reoxygenation. Cardiovascular Res. (2001) 51:265–74. 10.1016/S0008-6363(01)00294-2 11470466

[171] Streitner F Kuschyk J Veltmann C Brueckmann M Streitner I Brade J., Prospective study of interleukin-6 and the risk of malignant ventricular tachyarrhythmia in ICD-recipients–a pilot study. Cytokine. (2007) 40:30–34. 10.1016/j.cyto.2007.07.187 17851087

[172] Zhang Q Wang H Xue J Wu D. Associations between IL-6 variations and congenital heart disease incidence among chinese han people. Med. Sci. Monit. (2020) 26:e921032. 10.12659/MSM.921032 32519679 PMC7301674

[173] Wang D Fang J Wang R Sun D Xia K Yin K., Elevated serum ghrelin, tumor necrosis factor-α and interleukin-6 in congenital heart disease. Pediatr Int. (2016) 58:259–264. 10.1111/ped.12773 26256999

[174] Afify MF Mohamed GB El-Maboud MA Abdel-Latif EA. Serum levels of ghrelin, tumor necrosis factor-alpha and interleukin-6 in infants and children with congenital heart disease. J Tropical Pediatrics. (2009) 55:388–92. 10.1093/tropej/fmp036 19491251

[175] Zhou Y Pang B Xiao Y Zhou S He B Zhang F. The protective microRNA-199a-5p-mediated unfolded protein response in hypoxic cardiomyocytes is regulated by STAT3 pathway. J Physiology Biochemistry. (2019) 75:73–81. 10.1007/s13105-018-0657-6 30426460

[176] Heying R Qing M Schumacher K Sokalska-Duhme M Vazquez-Jimenez JF Seghaye MC. Myocardial cardiotrophin-1 is differentially induced in congenital cardiac defects depending on hypoxemia. Future Cardiology. (2014) 10:53–62. 10.2217/fca.13.99 24344663

[177] Kraśniak A Drozdz M Pasowicz M Chmiel G Michałek M Szumilak D., Factors involved in vascular calcification and atherosclerosis in maintenance haemodialysis patients. Nephrology, Dialysis, Transplantation. (2007) 22:515–521. 10.1093/ndt/gfl564 17050638

[178] Lee CT Chua S Hsu CY Tsai YC Ng HY Kuo CC., Biomarkers associated with vascular and valvular calcification in chronic hemodialysis patients. Disease Markers. (2013) 34:229–235. 10.1155/2013/846059 23396289 PMC3810241

[179] Roy N Rosas SE. IL-6 Is Associated with Progression of Coronary Artery Calcification and Mortality in Incident Dialysis Patients. Am J Neuro. (2021) 52:745–52. 10.1159/000518652 34535589 PMC8563392

[180] Kamińska J Stopiński M Mucha K Jedrzejczak A Gołebiowski M Niewczas MA. IL 6 but not TNF is linked to coronary artery calcification in patients with chronic kidney disease. Cytokine. (2019) 120. 10.1016/j.cyto.2019.04.002 30991230

[181] Kurozumi A Nakano K Yamagata K Okada Y Nakayamada S Tanaka Y. IL-6 and sIL-6R induces STAT3-dependent differentiation of human VSMCs into osteoblast-like cells through JMJD2B-mediated histone demethylation of RUNX2. Bone. (2019) 124:53–61. 10.1016/j.bone.2019.04.006 30981888

[182] Collin-Osdoby P. Regulation of vascular calcification by osteoclast regulatory factors RANKL and osteoprotegerin. Circulation Research. (2004) 95:1046–57. 10.1161/01.RES.0000149165.99974.12 15564564

[183] Deuell KA Callegari A Giachelli CM Rosenfeld ME Scatena M. RANKL enhances macrophage paracrine pro-calcific activity in high phosphate-treated smooth muscle cells: dependence on IL-6 and TNF-α. Journal Of Vascular Research. (2012) 49:510–21. 10.1159/000341216 22948607 PMC4001814

[184] Yokota K Sato K Miyazaki T Aizaki Y Tanaka S Sekikawa M. Characterization and function of tumor necrosis factor and interleukin-6-induced osteoclasts in rheumatoid arthritis. Arthritis Rheumatology (Hoboken, N.J.). (2021) 73:1145–54. 10.1002/art.41666 33512089 PMC8361923

[185] Lee GL Yeh CC Wu JY Lin HC Wang YF Kuo Yy., TLR2 Promotes vascular smooth muscle cell chondrogenic differentiation and consequent calcification via the concerted actions of osteoprotegerin suppression and IL-6-mediated RANKL induction. Arterioscler Thromb Vasc. (2019) 39:432–45. 10.1161/ATVBAHA.118.311874 30626205

